# Measuring Coverage in MNCH: New Findings, New Strategies, and Recommendations for Action

**DOI:** 10.1371/journal.pmed.1001423

**Published:** 2013-05-07

**Authors:** Jennifer Bryce, Fred Arnold, Ann Blanc, Attila Hancioglu, Holly Newby, Jennifer Requejo, Tessa Wardlaw, the CHERG Working Group on Improving Coverage Measurement

**Affiliations:** 1 Institute for International Programs, Department of International Health, The Johns Hopkins University, Baltimore, Maryland, United States of America; 2 MEASURE DHS, ICF International, Calverton, Maryland, United States of America; 3 Population Council, New York, New York, United States of America; 4 Statistics and Monitoring Section, Division of Policy and Strategy, United Nations Children's Fund, New York, New York, United States of America; Clinical Senior Lecturer in Maternal & Fetal Medicine and Honorary Consultant in Obstetrics, Women's Health Academic Centre, Kings College, London, United Kingdom

## Abstract

Considerable progress has been made in reducing maternal, newborn, and child mortality worldwide, but many more deaths could be prevented if effective interventions were available to all who could benefit from them. Timely, high-quality measurements of intervention coverage—the proportion of a population in need of a health intervention that actually receives it—are essential to support sound decisions about progress and investments in women's and children's health. The *PLOS Medicine* “Measuring Coverage in MNCH” Collection of research studies and reviews presents systematic assessments of the validity of health intervention coverage measurement based on household surveys, the primary method for estimating population-level intervention coverage in low- and middle-income countries. In this overview of the Collection, we discuss how and why some of the indicators now being used to track intervention coverage may not provide fully reliable coverage measurements, and how a better understanding of the systematic and random error inherent in these coverage indicators can help in their interpretation and use. We draw together strategies proposed across the Collection for improving coverage measurement, and recommend continued support for high-quality household surveys at national and sub-national levels, supplemented by surveys with lighter tools that can be implemented every 1–2 years and by complementary health-facility-based assessments of service quality. Finally, we stress the importance of learning more about coverage measurement to strengthen the foundation for assessing and improving the progress of maternal, newborn, and child health programs.

*Please see later in the article for the Editors' Summary*

## Abstract 2

Measuring Coverage in Maternal and Child Health: New Findings, New Strategies and Recommendations for Action In this overview of the PLOS Medicine Collection on “Measuring Coverage in Maternal and Child Health, Jennifer Bryce and colleagues discuss how and why some of the indicators now being used to track intervention coverage may not provide fully reliable measurements, draw together strategies proposed across the Collection for improving these measurements and make recommendations for action.

*This paper is part of the* PLOS Medicine *“Measuring Coverage in MNCH” Collection.*

## Introduction

Despite dramatic declines in child mortality over the past decade, in 2011 there were about 7 million deaths among children less than five years of age [1]. Moreover, declines have been slower for newborn [1] and maternal deaths [2]. Effective interventions are available to prevent most of these deaths [3], but are not reaching all those who need them. Global monitoring of the proportion of women and children in need of these interventions who actually receive them (referred to here as “intervention coverage”) shows both progress and missed opportunities to save lives [4]. Gaps in coverage are concentrated in poor countries, and within countries among the most vulnerable—the poorest and the least educated [5].

Monitoring of coverage levels for maternal, newborn, and child health (MNCH) interventions is central to assessing progress toward national and international health goals. Coverage data are also needed at national and sub-national levels to identify underserved populations and to monitor the effectiveness of strategies to reach them. The UN Secretary-General's global strategy, *Every Woman*, *Every Child*, calls for the scale-up of high-impact interventions, with oversight by an independent Expert Review Group. The independent Expert Review Group's 2012 report found that only 11 of the 75 countries that together account for over 95% of deaths among women and children had recent data on all eight coverage indicators recommended for global monitoring [6]. This finding reflects experience gained in tracking progress toward the Millennium Development Goals [7], and points to the challenges that must be overcome to improve the use of information for action. Examples of these challenges include obtaining adequate sample sizes for disaggregated analysis and reporting—and here new work on small area estimation techniques holds promise—and the need for temporally specific and recent measurements (i.e., data available within 12 months of collection).

Most data on intervention coverage in low- and middle-income countries are generated through the United States Agency for International Development-supported Demographic and Health Surveys (DHS) program [8], the United Nations Children's Fund-supported Multiple Indicator Cluster Surveys (MICS) program [9], and other national surveys modeled on these standards. DHS and MICS surveys send well-trained fieldworkers to interview preselected sample households (a probability sample in which each household has a known, non-zero chance of selection). Fieldworkers then conduct interviews with mothers and other caregivers about their health needs and interventions received as a basis for estimating coverage at the population level [10]. Household surveys—especially DHS and MICS surveys, which have refined their methods through more than 500 nationally representative surveys over more than two decades—are the primary tools available at present to track population-based trends in MNCH intervention coverage [11]. In most low- and middle-income countries, reports produced by routine health information systems include individuals in contact with the health system but miss those who are not and are often incomplete, late, or inaccurate. These reports do not, therefore, currently produce data adequate to support programmatic decisions, with occasional exceptions (e.g., in some countries with high vaccination coverage) [12]. Moreover, household surveys, unlike health information system reports, permit the analysis of coverage by equity variables such as gender, wealth, geography, and ethnicity.

Few studies have assessed the validity of coverage indicators for MNCH interventions measured through household surveys, or explored ways to improve their measurement. The aim of the *PLOS Medicine* “Measuring Coverage in MNCH” Collection is to bring together cutting-edge scientific work in this area. The Collection includes the results of a three-year program of research and reviews carried out by a diverse group of experts working under the auspices of the Child Health Epidemiology Reference Group (CHERG) [13], as well as work supported by other groups.

The CHERG work on improving coverage measurement began in 2009, guided by a Core Group that includes the technical directors of DHS and MICS and others with expertise in measurement issues (the named authors of this paper). We defined the scope of work (Box 1) and assessed the evidence base on the validity of coverage indicators for each proven intervention along the continuum of care for MNCH tracked by the Countdown to 2015 for Maternal, Newborn and Child Survival [14]. We established collaborative relationships with groups addressing measurement issues for particular indicators. For indicators with specific definitional or measurement issues that could be readily improved, we addressed them directly with the DHS and MICS leadership, and in most cases were able to resolve the problem. We prioritized indicators for which we were unable to find evidence that validity had been assessed, and commissioned research studies to assess the validity of these indicators and to identify ways to improve their measurement. Papers on methodological issues (e.g., uncertainty and interpretation [15] and equity [16]) were also commissioned.

**Box 1. Scope of the Collection**

The *PLOS Medicine* “Measuring Coverage in MNCH” Collection includes nine reviews, six research papers, and an overview of the measurement of population coverage for interventions of proven benefit to women's and children's health. It focuses on the following:

- Coverage measurement for interventions of proven efficacy that are feasible for implementation at scale in low- and middle-income countries to reduce maternal, newborn, and under-five mortality
- Global consensus coverage indicators as defined in the Countdown to 2015 for Maternal, Newborn and Child Survival
- Indicators that are or could be measured through population-based household surveys, with a specific focus on the core DHS and MICS surveys

We adopted the definition of coverage presented above (“the proportion of women and children in need of interventions who actually receive them”) in preference to measures of “effective coverage” that include estimates of intervention effectiveness, access, utilization, and service quality [17],[18]. Composite “effective coverage” metrics can have limited usefulness in national or global monitoring because they usually require a great deal of data that are rarely available in low-income countries and because they sometimes rely on modeling that produces “black box” statistics that must be unpacked to guide program decisions. Indeed, perhaps for these reasons, composite “effective coverage” metrics have generally not been adopted for use in monitoring progress toward global goals, including the Millennium Development Goals.

The Core Group oversaw the commissioned research studies, conceived of the Collection as a means of disseminating the findings, and invited groups working in related areas to contribute. Authors of the papers (the CHERG Working Group on Improving Coverage Measurement) met in Baltimore in June 2012 to review draft manuscripts and to ensure that the Collection addressed key themes and was internally consistent. Each manuscript was reviewed by at least two members of the Working Group and the Technical Deputy Director of DHS (Fred Arnold) prior to submission for publication. The full Collection is available at http://www.ploscollections.org/measuringcoverageinmnch.

The Collection presents new evidence drawn from research studies and reviews of existing literature. In this overview we synthesize the findings of the Collection papers under three broad topics: (1) validity of coverage estimates based on respondents' reports, (2) potential strategies for improving coverage measurement through household surveys, and (3) crosscutting methodological issues in coverage measurement. We close with a set of action recommendations directed to those who conduct, support, or use household survey results on measurement of coverage for MNCH interventions.

## The Validity of Coverage Estimates Based on Respondents' Reports

At the core of the Collection is a set of research studies that assesses the validity of respondents' reports on interventions that they and the children under their care have received. The basic design of these studies was to first establish an accurate record of the need for interventions (e.g., standard or emergency obstetric services, treatment for pneumonia or malaria), and then to either conduct direct observations in health facilities or obtain high-quality health records (the “reference standard”) to determine which interventions were provided to address this need. The next step involved visiting each woman in her community to administer survey questions using time intervals and question sequences similar to those in the DHS and MICS household surveys. These data were then used to estimate the sensitivity, specificity, and accuracy of the mothers' individual reports, and to determine whether indicator values would be overestimated or underestimated when measured in a household survey (the true/actual positives ratio or its mathematical equivalent) [19]. This design may produce results that are positively biased for interventions that can be offered either at home or at a health facility because the samples are drawn from health service settings. However, even studies based on determinations of need conducted in purposively selected teaching hospitals offer important evidence about the research question on mothers' recall of health-related events.

We conducted studies of this type for emergency cesarean sections in Ghana and the Dominican Republic [20], a broad range of interventions delivered around the time of birth (the peripartum period) in Mozambique [21], diagnosis and antibiotic treatment of childhood pneumonia in Pakistan and Bangladesh [22], diagnosis and treatment of malaria in Zambia [23], and selected services across the MNCH continuum of care in a rural population in China [24]. Table 1 presents findings on sensitivity, specificity, and accuracy for a subset of the indicators assessed in the research studies, focusing on global consensus coverage indicators currently in use and indicators closely related to these accepted coverage indicators. These results are not strictly comparable across studies owing to differences in what is being measured, how it is being measured, and in what context. We believe for the same reason that applying standard cutoff values for acceptable levels of accuracy is not appropriate in this situation. True/actual positives ratios or their mathematical equivalents are presented in the individual papers [20],[21],[23],[24].

**Table 1:** Selected Collection research findings on respondents' reports of intervention coverage.

Category	Intervention (Selected)	Collection Research Paper		Selected Findings		
		Total Study Sample^a^ (Reference Standard)	Reference	Sensitivity (CI)	Specificity (CI)	Accuracy/AUC (CI)
**Antenatal care**	First antenatal care visit <12 weeks of gestational age	914 women aged 18–49 in China with at least one live birth in the preceding five years (home-based booklets and electronic service records)	Guo, et al. [24]	90% (86–94)	22% (19–26)	56% (54–59)
	At least four antenatal care visits for last pregnancy	Same as previous		98% (96–99)	25% (19–32)	62% (58–65)
**Interventions delivered around the time of birth**	Woman delivered in a hospital (versus a health center)	304 women in Mozambique who gave birth 8–10 months previously in government facility (direct observation by trained clinician)	Stanton, et al. [21]	81% (75–87)	94% (90–98)	88% (84–91)
	Newborn placed skin-to-skin on mother's chest	Same as previous		60% (52–69)	69% (62–76)	65% (59–70)
	Newborn immediately dried	Same as previous		77% (72–82)	31% (12–50)	54% (45–63)
	Mothers' recall of emergency cesarean section^a^	659 women in Ghana delivered in a hospital via cesarean section (facility-based data supplemented by information requested from the medical staff)	Tunçalp, et al. [20]	79% (73–83)	82% (78–85)	80% (77–83)
		1,531 women in the Dominican Republic delivered in a hospital via cesarean section (facility-based data supplemented by information requested from the medical staff)		50% (47–53)	80% (77–83)	65% (62–67)
	Any cesarean section	914 women aged 18–49 in China with at least one live birth in the preceding five years (home-based booklets and electronic service records)	Guo, et al. [24]	96% (93–99)	83% (80–86)	90% (88–92)
**Vaccination**	Diphtheria-tetanus-pertussis vaccine	914 women aged 18–49 in China with at least one live birth in the preceding 5 years (home-based booklets and electronic service record)	Guo, et al. [24]	89% (86–92)	70% (61–78)	80% (75–84)
	Measles vaccine	Same as previous		95% (92–98)	44% (38–49)	69% (66–72)
**Treatment of childhood illness**	Correct treatment of pneumonia (using DHS algorithms on symptoms of acute respiratory infection and detailed enquiry)	672 caregivers of children 0–59 months diagnosed pneumonia or “no pneumonia” in the out-patient department of an urban hospital in Islamabad, Pakistan (direct observation)	Eisele, et al. [22]	67% (62–72)	69% (64–74)	0.66 (0.62–0.69)
		700 caregivers of children 0–59 months diagnosed pneumonia or “no pneumonia” in the out-patient department of an urban hospital in Dhaka, Bangladesh (direct observation)		24% (19–30)	82% (77–87)	0.53(0.49–0.57)
		478 caregivers of children 0–59 months diagnosed pneumonia or “no pneumonia” in rural Mirzapur, Bangladesh (direct observation)		72% (65–78)	55% (47–62)	0.63 (0.59–0.68)
	Child with fever	601 caregivers at least 18 years old of children under five years old presenting for treatment for fever in five health centers in the previous two weeks, Zambia (recording by trained clinician)	Eisele, et al. [23]	96% (87–100)	100% (—)	96% (87–100)
	Finger/heel stick performed	Same as previous		63% (18–100)	90% (86–94)	72% (41–100)
	Malaria diagnosis made	Same as previous		77% (55–99)	76% (48–100)	76% (55–98)
	Artemisinin combination therapy given	Same as previous		81% (51–100)	92% (80–100)	85% (73–98)

a This is the total number of completed interviews with respondents; sample sizes for specific indicators may be smaller and readers are referred to the original article.

AUC, area under the receiver operating characteristic curve; CI, confidence interval.

The studies show that the sensitivity and specificity of coverage indicators are highly variable across interventions, with some suggestion of more accurate reporting for events related to care-seeking behaviors (e.g., place of delivery) or invasive interventions (e.g., finger/heel stick or cesarean section performed). Women reported less accurately about interventions that occurred immediately after childbirth, such as whether the newborn was dried [21], or for interventions requiring recognition of a complex disease syndrome such as pneumonia [22]. Also noteworthy are large differences in the sensitivity and specificity of individual indicators within and across countries, such as urban–rural differences in Bangladesh [22], and in the sensitivity of several indicators in different settings [20],[22]. The findings of the studies must be generalized with caution, however, because the sensitivity and specificity of the indicators may vary by characteristics of the host and the pathogen, the broader epidemiological setting (e.g., low levels of falciparum malaria in Bangladesh and Pakistan), and cultural and educational differences in interpreting and responding to survey questions.

Another set of challenges is more structural, and relates to the difficulty of measuring coverage using respondents' reports for interventions that address relatively rare events. Even if mothers can report accurately, obtaining adequate denominators to support coverage measurement for low prevalence events requires very large samples—as does the determination of reasonably precise disaggregated estimates—and, for some indicators, requires attention to be paid to seasonality issues [25].

The research paper on using community surveys to estimate HIV survival [26] uses a different design, but makes an important contribution to the Collection as a whole. The authors demonstrate that in four African countries (Cameroon, Côte D'Ivoire, South Africa, and Zambia), health-facility-based estimates of coverage for regimens to prevent mother-to-child HIV transmission consistently tend to overestimate true population coverage measured in the community. The authors also show that the health-facility-based estimates do not correlate well with infant HIV-free survival. These results serve as a wake-up call to those advocating for coverage measurement based solely on routine health-facility-based information systems.

Based on the research findings in this Collection, we can make concrete recommendations about which indicators perform well in household surveys, about which perform poorly and may produce spurious results, and about whether there are new indicators that are good candidates for inclusion in future surveys. Indicators that performed well in this set of validation studies include place of delivery (hospital, health center, home), placing the newborn skin-to-skin against the mother's chest, emergency cesarean section, and treatment of childhood malaria with artemisinin-based combination therapies. Coverage indicators for antibiotic treatment of childhood pneumonia showed poor results [22],[25], consistent with previous research indicating that lists of illness signs are poor predictors of actual disease [27]–[31]. For malaria, presumptive treatment of fever with antimalarials is no longer recommended; malaria treatment should be given only to children with a malaria diagnosis confirmed by microscopy or rapid diagnostic test, where possible. Ideally the global indicator for malaria treatment would reflect this policy and be limited to children with laboratory-confirmed disease, but the relatively poor accuracy of correct recall of a malaria diagnosis [23] currently precludes this change.

These research results are buttressed and extended by the findings from the Collection reviews of previous research on the accuracy of respondents' reports of services received. Many factors can affect the reliability of a verbal history of service provision, including the information received or understood at the time the intervention was delivered, interviewer behaviors, the recall period, the characteristics and salience of the intervention itself, and the length of the questionnaire and resulting interview fatigue [32]–[35]. Previous studies have shown, for example, that women have difficulty reporting on interventions provided during labor and in the first hours after birth, especially in surveys asking about these events up to two years after they occur [36]. Similarly, although an early household survey in Mozambique found that mothers' reports of symptoms of dehydration in the last 24 hours among children with diarrhea corresponded reasonably well with the diagnosis of dehydration by trained interviewers, the Collection review on intervention coverage for diarrhea identifies challenges in the measurement of diarrhea treatment coverage that arise from difficulties in standardizing definitions of diarrhea severity and a lack of clarity about the types and quantities of fluids required to treat diarrhea [37].

## Potential Strategies for Improving Coverage Measurement through Household Surveys

The papers in this Collection propose many ways in which household surveys might be improved to produce more valid estimates of intervention coverage. We review them briefly here and direct readers to the full papers for further explanation.

### Use Aides Mémoires to Improve Accurate Reporting

Hazir and colleagues in Pakistan and Bangladesh used expanded lists of clinical signs of pneumonia and video clips of children with specific clinical presentations in caregiver interviews to increase the accuracy of the denominator for the indicator on antibiotic treatment of pneumonia, and showed mothers a selection of locally available antibiotics to increase the accuracy of the numerator [22]. Their results were mixed. The video showed much higher sensitivity in Pakistan (where it was developed) than in Bangladesh (62% versus 28%), whereas the use of visual prompts for antibiotic treatment, a common practice in DHS and MICS surveys, was associated with increased accuracy of reports of treatment regimens in both settings. Implementation of this strategy may be challenging, however, in settings with an active private sector and many potential alternative sources of medicine. Small-scale trials of similar techniques may yield important findings about how recall accuracy can be improved for interventions that address a broader range of conditions.

### Refine the Survey Questionnaire and/or Procedures

The papers in this Collection suggest that attention should be refocused away from indicators that are not producing valid measurements to those that do, even if these alternative indicators do not provide a full answer to the question about whether a specific intervention was received. For example, indicators related to whether care was sought for a child with signs of pneumonia—although not yet validated—offer promise as measures of access to correct pneumonia treatment and could be coupled with assessments of correct management collected in health service sites [25]. Another way forward might be to assess care seeking for children with signs of any illness, avoiding the pitfall of constructing differential diagnoses based on respondent reports. Alternative question formulations also hold promise for improved maternal reporting on interventions received around the time of birth [20],[21]. Moreover, previous research on cognitive aspects of recalling health-related events in developed country settings (e.g., [38]) may hold additional untapped potential for improving MNCH coverage measurement in low- and middle-income countries.

In their papers, the Collection authors make numerous other suggestions for changes in the core DHS/MICS questionnaires and/or procedures. Some are relatively easy to implement and have already been adopted based on preliminary findings. For example, core questionnaires for DHS and MICS surveys now include response options for questions about where a child was taken for care that include the workers responsible for providing community case management [39]. Other suggestions are more ambitious, such as adding questions on the severity of diarrhea [37] or adding HIV testing of mothers and infants in more surveys [26].

Several of the Collection studies report new findings that compare the accuracy of mothers' reports of intervention receipt for different recall periods. Understanding the effect of the recall period on the accuracy of reports is important because where longer recall periods do not jeopardize accuracy by introducing bias they can yield an increased sample size for the measurement of specific indicators without increasing the number of households surveyed. Hazir et al. found no differences in the accuracy of mothers' reports related to childhood pneumonia at two and four weeks [22], which suggests that in future surveys more children with signs of pneumonia can be included in the estimation of indicators for care seeking, if not treatment. Eisele et al. found no drop-off in the accuracy of recalling malaria diagnosis and treatment interventions two weeks versus one week after the clinic visit [23]. These results warrant confirmation, as earlier work has suggested that reporting accuracy decreases with longer recall periods for duration of breastfeeding [40],[41], signs of respiratory illness in children [27],[42], and a broader range of health symptoms [43].

### Link Household Surveys to Other Sources of Information about Service Provision

Our understanding of services received by mothers and children can be improved by linking information on the sources of care obtained through household surveys to facility assessments of the extent and quality of the interventions delivered. In this way, for example, coverage estimates for peripartum interventions that women who report delivering in a health facility are unable to recall could be linked to temporally specific information about the standard practices and quality of care at that facility. This approach was used in an evaluation in Bangladesh that linked detailed information on care seeking for childhood illness obtained from mothers during a household survey with observation-based assessments of the quality of child health care offered by those providers [44]. Similarly, vaccination records held at health facilities can be sought when a home-based record is not available [32]. The malaria research community is also considering the use of a set of indicators that measure aspects of treatment-seeking practices, diagnosis, and treatment from a combination of sources including household and health facility surveys, rather than the single indicator of receipt of artemisinin combination therapy among children with fever [45]; results presented in this Collection provide further justification for that decision [23]. Tools for assessing service provision [46] and quality of care [47]–[49] exist for most MNCH interventions; the methodological challenge is to link them to household surveys in ways that produce valid population-based coverage estimates at reasonable cost.

### Incorporate Information Technology

As digital technology becomes more widespread, the potential for quick and accurate electronic recording and transmission of intervention data will improve rapidly. Digital registries offer opportunities to improve monitoring of target populations and of interventions, such that accurate denominators and numerators will become increasingly feasible to obtain via routine administrative data in low- and middle-income countries. Advances in the use of electronic and telecommunications processes in health (e-health) and the use of mobile devices to collect health information in surveys and patient care (m-health) also have important implications for coverage measurement and monitoring. Finally, technology can improve data quality in household surveys and is increasingly being used in real-time measurement of child health program indicators (e.g., [50]). However, further research and evaluation is needed, with strong coordination, to develop and roll out the effective use of digital records and to avoid a proliferation of different, often incompatible, e-health projects.

### Increase the Salience of Intervention Delivery

Health personnel may also be able to contribute to improved coverage measurement. Health workers should explain to mothers the importance of keeping the home-based child health or vaccination record carefully and bringing it to every health facility visit. Reinforcement of the importance of home-based records should increase their availability during household surveys. Careful explanations by service providers about the interventions being delivered, and why, are also likely to increase recall. Explanations need not be limited to the patient contact during which the service is provided. For example, telling a pregnant woman during an antenatal care visit that she should receive an injection immediately following birth may increase her recall of the event. In addition, the salience of intervention delivery may be increased by having an outreach or community health worker wear a highly visible, unique article of clothing (e.g., community health workers providing case management to sick children in Bangladesh carried a bright pink bag to help mothers differentiate them from other types of community workers). Advance planning and creativity on the part of program personnel can yield benefits for coverage monitoring.

### Use Measures That Do Not Rely on Respondents' Reports

A final strategy for improving coverage measurement is to find ways to replace mothers' recall with more objective measures. The use of home-based records of services received offers an attractive alternative to mothers' recall, if the records contain complete and accurate information. However, the review of experience with home-based vaccination records conducted as part of this Collection highlights multiple potential sources of error, including incomplete, inaccurate, or outdated records, as well as errors in transcription [32]. A review of DHS and MICS surveys since 2000 found that home-based vaccination records were available for less than 70% of children in 21 of the 33 least developed countries [51]. The report from China in this Collection, which used a combination of home-based records and an electronic system as the reference standard, had high proportions of missing data for all the coverage indicators assessed [24].

It may also be possible to expand the use of examinations and biological testing to assess coverage for certain indicators and to generate evidence that an intervention has actually been received. Although experience to date has largely been limited to testing for the presence of infection during a household interview (e.g., rapid diagnostic tests for anemia and malaria and testing of mothers and infants for HIV antibodies), work has begun on methods to determine whether specific medicines have been received, and there is growing interest and experience in using medically trained teams to conduct household surveys that include physical examinations and the collection of biological specimens [52]. Measurement of tetanus and measles antibodies in serum or oral fluid to determine receipt of diphtheria-tetanus-pertussis and measles vaccines is also feasible, although each currently has caveats [32]. The potential of such measures and their practicality and cost should be explored.

## Crosscutting Methodological Issues in Coverage Measurement

Three methodological themes appear consistently throughout the Collection papers. First, household surveys based on probability sampling, and conducted with careful attention to quality in sampling, interviewer training, fieldwork, data management and cleaning, and analysis are the bedrock of coverage monitoring at the population level. Although there may be cheaper alternatives to conducting high-quality, nationally representative surveys to ascertain intervention coverage, the use of these alternatives should be treated with caution unless the same rigor is used in them for sampling and quality control as in the larger surveys [15],[32]. Facility-based assessments are not based on probability samples of the population, will often overestimate population coverage [26], and cannot replace household surveys for the measurement of intervention coverage as defined here.

Second, no matter how carefully intervention coverage is measured through household surveys, sampling and non-sampling error is always present and must be accounted for when interpreting results for decision-making and program evaluation purposes [15]. To ensure that sampling error is considered, we recommend that all surveys publish appropriately calculated confidence intervals for key coverage indicators, as is already done in DHS and MICS reports, and refer to them consistently when presenting and interpreting the results. Countries implementing nonstandard surveys should ensure that they measure global coverage indicators comparable to those produced by DHS and MICS, and reports should describe the sampling design in sufficient detail to allow determination of whether a true probability sample was drawn and whether the survey design was appropriately accounted for in the calculation of the standard errors. To account for non-sampling error such as information and selection bias, survey reports should also include a limitations section that explicitly lists probable sources of non-sampling error, and authors should speculate about the direction and magnitude of error where possible.

Third, disaggregated reporting makes data on intervention coverage more useful to policy and program decision makers. Disaggregation of national coverage estimates by wealth, geographic region, and other relevant stratifiers helps identify groups that are not being reached. A paper in this Collection provides basic guidance on the measurement and interpretation of inequalities in health coverage data from household surveys [16]. As targets are set for universal health coverage, governments and their partners must actively seek out and demand data that help them develop effective, local delivery strategies to reach those who are currently not receiving services, and must seek ways to generate community demand for essential MNCH services.

## Action Recommendations

We hope this Collection will serve as a vehicle for advancing the field of intervention coverage measurement in maternal and child health by providing a strong justification for increased attention to the quality and precision of coverage estimates. One aim of this Collection is to inform those who use coverage indicators at global and national levels, so that they can make sound choices about the selection of indicators and can interpret the results intelligently by recognizing their uncertainty bounds [15],[53]. A second aim is to highlight actions needed by care providers and researchers in the MNCH community to improve coverage measurement and the use of coverage results (Box 2).

**Box 2. What Needs to Be Done to Improve MNCH Coverage Measurement?**

- Efforts to conduct high-quality household surveys at national and sub-national levels must be sustained to provide essential information on coverage trends and inequities, even as routine health information systems improve.
- These large surveys need to be supplemented with lighter tools that can be implemented every 1–2 years to produce high-quality estimates of MNCH intervention coverage.
- Further investment is needed in complementary assessments of service quality in health service settings, including the delivery of specific interventions during service contacts; these assessments should be synchronized in time and linked geographically to population-based household surveys that measure coverage for the same interventions.
- Efforts to learn more about coverage measurement using innovative designs to assess validity must be recognized and supported.

High-quality household survey programs will continue to be the primary source of data on MNCH intervention coverage for the foreseeable future, even as routine health information systems improve. These surveys are needed to validate and calibrate data produced from other sources, to investigate variations in coverage in specific subgroups, and to assess equity by gender or income. Careful thought needs to be given to how we can ensure household surveys continue to produce the best and most relevant information that is needed by public health decision makers. The survey protocols must be continuously adapted to capture new interventions and delivery strategies for which coverage is not currently measured and to incorporate new evidence about coverage measurement, such as that presented in this Collection. Supplemental survey tools that are tailored to the need for more frequent measurement of MNCH intervention coverage may be required as country and global interest in accountability grows. These supplemental survey tools might focus on what surveys do well—the assessment of coverage for interventions that are clearly defined and highly salient (the numerator) and needed by all members of a specific population subgroup (the denominator)—and leave more challenging measurements for the full and more complex surveys every 3–5 years.

Methodological work is also needed to link survey data on sources of health care to rigorous, comparable assessments of the extent and quality of interventions being delivered in those settings. As routine health information systems improve, especially in middle-income countries, there may be opportunities to calibrate them with data collected from representative samples of the population to increase their usefulness in coverage measurement.

Perhaps the most important message of this Collection is that much remains to be learned about how best to measure MNCH intervention coverage through household surveys. The questions are clear and can be answered through well-designed studies building on and extending the work started here. Options for improving existing metrics need to be systematically evaluated, including alternative question formulations, strategies to aid recall, and use of biomarkers and technologies. New indicators and modules, including those for neonatal interventions [34] and postnatal visits [35], need to be validated, as do indicators for additional intervention areas such as young child feeding that are not addressed in this Collection. Existing findings need to be confirmed among more representative populations in additional countries. Demographic surveillance sites might be engaged to conduct further tests, recognizing that they are not always representative of larger populations and may not currently be able to link household survey data with those generated through facility assessments. This learning agenda must be implemented urgently as a foundation for producing better evidence for stronger programs and improved accountability.

**Key Points**

- Regular, high-quality measurement of the proportion of women, newborns, and children in need of life-saving interventions who actually receive them (“intervention coverage”) is essential to support sound decisions at local, national, and global levels.
- Standardized household surveys based on probability sampling are the cornerstone of coverage monitoring and provide a wealth of important background information to support interpretation and equity analyses.
- Some of the indicators now being used to track intervention coverage may not provide fully accurate or reliable results for a variety of reasons, among them limitations of respondent recall and of using symptoms as a basis for defining specific diseases.
- A better understanding of the systematic and random error inherent in these coverage indicators—and approaches to mitigate that error—can help in indicator interpretation and use.
- Measurement of intervention coverage can be improved through focused operational research on household survey techniques, supplemented by more frequent assessments using less sophisticated or routine methods, and extended through links to assessments of service quality.

## Abbreviations

CHERG: Child Health Epidemiology Reference Group
DHS: Demographic and Health Survey/s
MICS: Multiple Indicator Cluster Survey/s
MNCH: maternal, newborn, and child health
